# Ga Based Particles, Alloys and Composites: Fabrication and Applications

**DOI:** 10.3390/nano11092246

**Published:** 2021-08-30

**Authors:** Zhi Li, Yiming Guo, Yufen Zong, Kai Li, Shuang Wang, Hai Cao, Chao Teng

**Affiliations:** 1 Institute of Marine Biomedicine, Shenzhen Polytechnic, Shenzhen 518055, China; lizhireal@163.com (Z.L.); 20190286@szpt.edu.cn (K.L.); wangshuang20201009@163.com (S.W.); 2 National Laboratory of Solid State Microstructures, College of Engineering and Applied Sciences, Nanjing University, Nanjing 210093, China; 3 State Key Laboratory of Materials-Oriented Chemical Engineering, College of Chemical Engineering, Nanjing Tech University, Nanjing 211816, China; gym1395639936@163.com (Y.G.); 15540877426@163.com (Y.Z.)

**Keywords:** liquid metal, gallium-based materials, wearable electronics, energy storage and harvesting devices, bio-applications

## Abstract

Liquid metal (LM) materials, including pure gallium (Ga) LM, eutectic alloys and their composites with organic polymers and inorganic nanoparticles, are cutting-edge functional materials owing to their outstanding electrical conductivity, thermal conductivity, extraordinary mechanical compliance, deformability and excellent biocompatibility. The unique properties of LM-based materials at room temperatures can overcome the drawbacks of the conventional electronic devices, particularly high thermal, electrical conductivities and their fluidic property, which would open tremendous opportunities for the fundamental research and practical applications of stretchable and wearable electronic devices. Therefore, research interest has been increasingly devoted to the fabrication methodologies of LM nanoparticles and their functional composites. In this review, we intend to present an overview of the state-of-art protocols for the synthesis of Ga-based materials, to introduce their potential applications in the fields ranging from wearable electronics, energy storage batteries and energy harvesting devices to bio-applications, and to discuss challenges and opportunities in future studies.

## 1. Introduction

Liquid metals (LMs) are metals that can stay as a liquid near room temperature because of their low melting points, such as mercury (Hg, −38.8 °C), francium (Fr, 27 °C), cesium (Cs, 28.5 °C), rubidium (Rb, 39.3 °C) and gallium (Ga, 29.8 °C) [1]. Despite their excellent electrical, thermal properties and the unique fluidic state, most of LMs are of limited utility due to their unfriendly physicochemical nature. For example, Hg is highly toxic and easily evaporable, Fr is radioactive and Cs and Rb are instable in air. As a consequence, Ga emerges as the best choice in the LM catalog owing to its low toxicity and high chemical stability. Furthermore, a self-limited, native, thin oxide layer can be formed on the surface of Ga, which can be used as a shell for the formation of geometrically defined shapes of Ga-based nanoparticles with controllable size and properties or a nucleation site for further polymerization reaction. In order to widen the application range, Ga based eutectic alloys such as the commercially available eutectic gallium–indium–tin alloy (Galinstan or EGaInSn, 68.5 wt% Ga, 21.5 wt% In and 10.0 wt% Sn) and gallium–indium alloy (EGaIn, 78.6 wt% Ga and 21.4 wt% In) have been developed and upon varying their composition the resulting melting point can be adjusted below room temperature. In particular, Galinstan has been used, for example, as a coolant [2,3,4], a soft electronic material [5,6] and a lubricant [7,8]. EGaIn has been widely used in many areas, such as non-destructive contact in large-area junctions [9,10,11], nanomedicine carriers in drug delivery systems [12,13] and soft robotics [14,15,16]. To further enrich their functionalities, LM composites have been synthesized to build new material systems in which LMs/alloys are suspended as nanodroplets within a soft polymer matrix or mixed with other inorganic nanoparticles. We limit this review to the metals that are stable as liquids at or near the room temperature, while we exclude Hg, Fr, Cs and Rb due to the above-mentioned reasons. Therefore, the focus of LM in this review is on Ga and their alloys. We briefly summarize the latest development in the synthetic approaches for Ga based materials including Ga particles, alloys and their composites with polymers or rigid inorganic particles and their applications in such as soft electronics, energy storage and harvesting systems and bio-applications.

## 2. Synthesis of LM Based Materials

### 2.1. LM Droplets/Particles

**Figure 1:**
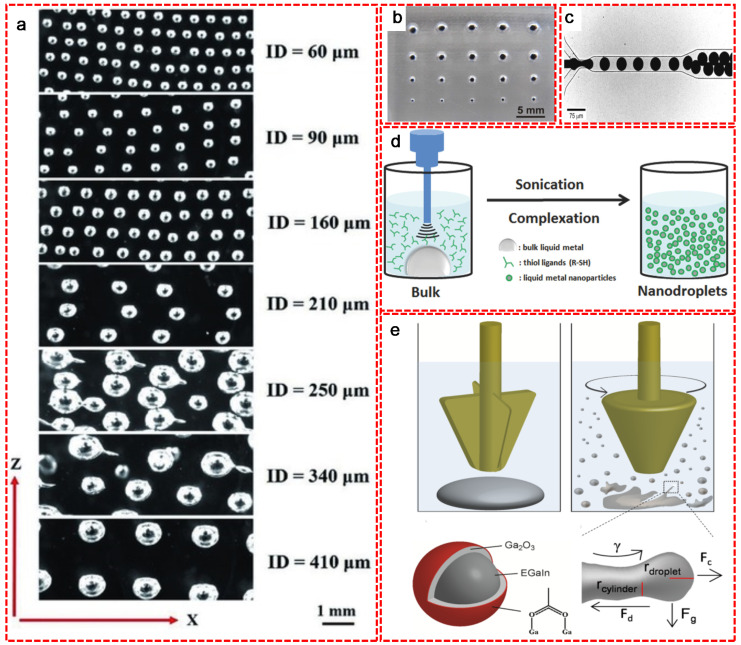
LM droplets/particles fabricated with various methods. (**a**) LM droplets with different sizes (ranging from 60 to 410 µm) produced by 3D printing method. Reproduced with permission. [17] Copyright 2017, Wiley-VCH. (**b**) Template-assisted molding to produce LM particles with different sizes. Reproduced with permission. [18] Copyright 2014, MDPI. (**c**) LM droplets produced in the flow-focusing microfluidic device with uniformly controlled size distribution. Reproduced with permission. [19] Copyright 2012, Wiley-VCH. (**d**) LM nanodroplets formed via sonication. Reproduced with permission. [20] Copyright 2016, Wiley-VCH. (**e**) Schematic description of the SLICE method to prepare LM particles in micro/nano scale, where γ, Fc, Fg and Fd indicate the shear, centrifugal, gravity and drag forces, respectively. Reproduced with permission. [23] Copyright 2014, American Chemical Society.

Over the past decades, LM droplets/particles have been produced by many fabrication methods (e.g., 3D printing, microfluidic approach, ultrasonication and molding) to control the droplet size and distribution. Figure 1 summaries the commonly used fabrication methods of LM droplets/particles.

**Figure 2:**
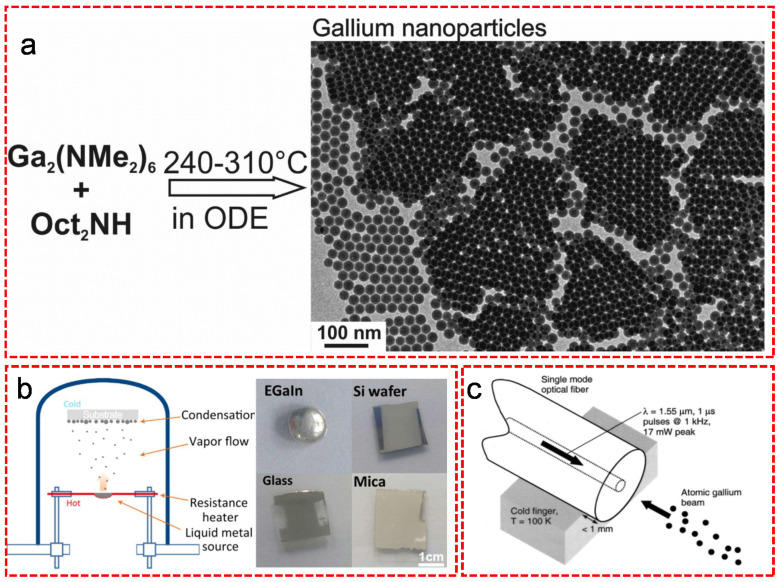
(**a**) The schematic of the synthesis of Ga nanoparticles via thermal decomposition of Ga-alkylamides. Reproduced with permission. [24] Copyright 2014, American Chemical Society. (**b**) The schematic for preparation of EGaIn nanoparticles on various substrates via physical vapor deposition. Reproduced with permission. [25] Copyright 2018, Elsevier B.V. (**c**) Sketch of the apparatus for preparation of gallium nanoparticles on the silica fiber from atomic gallium beam. Reproduced with permission. [26] Copyright 2002, American Institute of Physics.

As one of the most powerful additive manufacturing technologies, 3D printing has shown its ability in a wide range of newly emerging areas. Owing to its fluidic nature, LM based materials can work as new printable inks in an effortless and low-cost way. Yu et al. used self-healing hydrogel as the support medium to create macroscopic structures of LM that exhibited properties indicative of a non-printable object [17]. The size of the LM droplets ranging from 60 to 410 micrometers (Figure 1a) was accurately controlled by varying the inner diameter of nozzle, the flow rate and the printing speed. Moreover, molding is another effective way to fabricate LM droplets [17]. In this process, the raw material of LM is pressed into a rigid mold and the native oxide skin is removed by acid vapor. The size of LM droplets is easily controlled by the dimensions of reservoir. Mohammed et al. demonstrated a simple method of producing LM spheres by spreading EGaIn across an elastomeric sheet featuring cylindrical reservoirs defined by replica molding with diameters ranging from hundreds of microns to a couple of millimeters (Figure 1b) [18]. Additionally, the microfluidic approach is also a common method for the fabrication of LM droplets. Figure 1c briefly shows the fabrication process of LM droplets with a diameter < 30 μm in a microfluidic device with a 20 μm nozzle size in aqueous polyethylene glycol solution and silicone oil (as high viscosity fluids) [19]. The size of the monodisperse spherical micro-droplets mainly depends on the channel dimensions and the flow rate. In order to obtain multiple functions, LM droplets can be fabricated into nanoparticles via a simple but practical ultrasonication approach in the presence of ligands and organic solvents. The mechanism is illustrated in the Figure 1d [20]. The bulk LM was under ultrasonication treatment and split into small pieces in the presence of thiol ligands, which stabilized those small LM fragments to form nanoparticles. In the earlier research, Hohman et al. reported that the synthesis of EGaIn nanoparticles were performed by ultrasonication of bulk EGaIn in the presence of thiol ligands such as 1-dodecanethiol (C12) or 3-mercapto-N-nonylpropionamide (1ATC9) [21]. Fast thiolate self-assembly at the EGaIn interface protected the material against oxidation during the ultrasonic dispersion of the bulk EGaIn. Strong ligand–metal interaction induced surface strain and promoted large particles cleavage to nanoparticles. Finkenauer et al. systematically studied the stabilization effect of thiols with different alkyl chains and revealed that 1-octadecanethiol was the most efficient thiol for offering the smallest size and highest yield of eutectic EGaIn nanodroplets [22]. Similar to ultrasonication, shearing is also an efficient method to break bulk LM into small particles with diameters ranging from a few nanometers to few micrometers by changing the shearing speed and solvent. Tevis et al. reported a simple method called shearing liquid into complex particles (SLICE) that used emulsion shearing with EGaIn native oxide layer to make core-shell particles, and emulsion shearing with surface-tension driven phase segregation to synthesize particles with complex surface compositions and morphologies (Figure 1e) [23]. During this process, the bulk LM droplet was stretched by the shearing force into a cylinder-like shape that subsequently broke into small droplets upon reaching the Rayleigh-Plateau limit. Afterwards, the combined force from shearing, centrifugal, gravity and drag forces continued to split the droplets. Although the sonication and SLICE methods are efficient for massive production of small size LM particles, the uniformity of the particle size distribution is hard to control. This hinders the LM in some delicate fields such as optics or biologics. Therefore, some size-controlled fabrication methods have been applied for the synthesis of LM particles. Thermal decomposition of organometallic compounds is one of the representative size-controlled methods to synthesize LM particles with uniform size distribution. Yarema et al. prepared Ga nanoparticles with the average size tunable in the range of 12–46 nm and with excellent size distribution as small as 7–8% by thermal decomposition of the mixture of gallium tris(dimethylamide) dimer and di-n-octylamine in 1-octadecene at 240–310 °C (Figure 2a) [24]. Different from the thermal decomposition method that requires no substrate, vacuum based deposition and molecular beam epitaxy methods allow the deposition of Ga particles on various substrates. Yu et al. demonstrated that surfactant-free EGaIn nanoparticles were successfully synthesized with controllable particle size on various substrates via physical vapor deposition method (Figure 2b) [25]. For the deposition time at 100 s, the LM started to nucleate in the form of near-monodispersed small particles with a diameter of around 25 nm. Upon increasing the deposition time up to 300 s, the nuclei grew into middle size particles with an average diameter of around 70 nm, which merged into bimodal distributed large particles with an average diameter larger than 100 nm upon 500 s deposition. MacDonald et al. [26] and Fedotov et al. [27] exposed the silica fiber/substrates to the atomic gallium beam to fabricate gallium nanoparticles (Figure 2c). Controlling the size, shape and spatial distribution of the nanoparticles were achieved by manipulating the deposition conditions (e.g., atomic beam flux, substrate temperature) and laser excitation parameters (e.g., wavelength, power).

The particle size and distribution are vital for certain applications such as biomedicines. Particles formed by ultrasonication, thermal decomposition, physical vapor deposition and atomic Ga beam have a smaller size ranging from 10 to 100 nm, while other methods such as (e.g., 3D printing, template-assisted molding) normally generate micron-scale particles. Post-treatment (e.g., filtering) is helpful to obtain Ga particles with controlled homogeneous distribution, which is of great importance for the improvement of performance and safety on those sensitive applications with particle size.

### 2.2. LM-Polymer Composites

**Figure 3:**
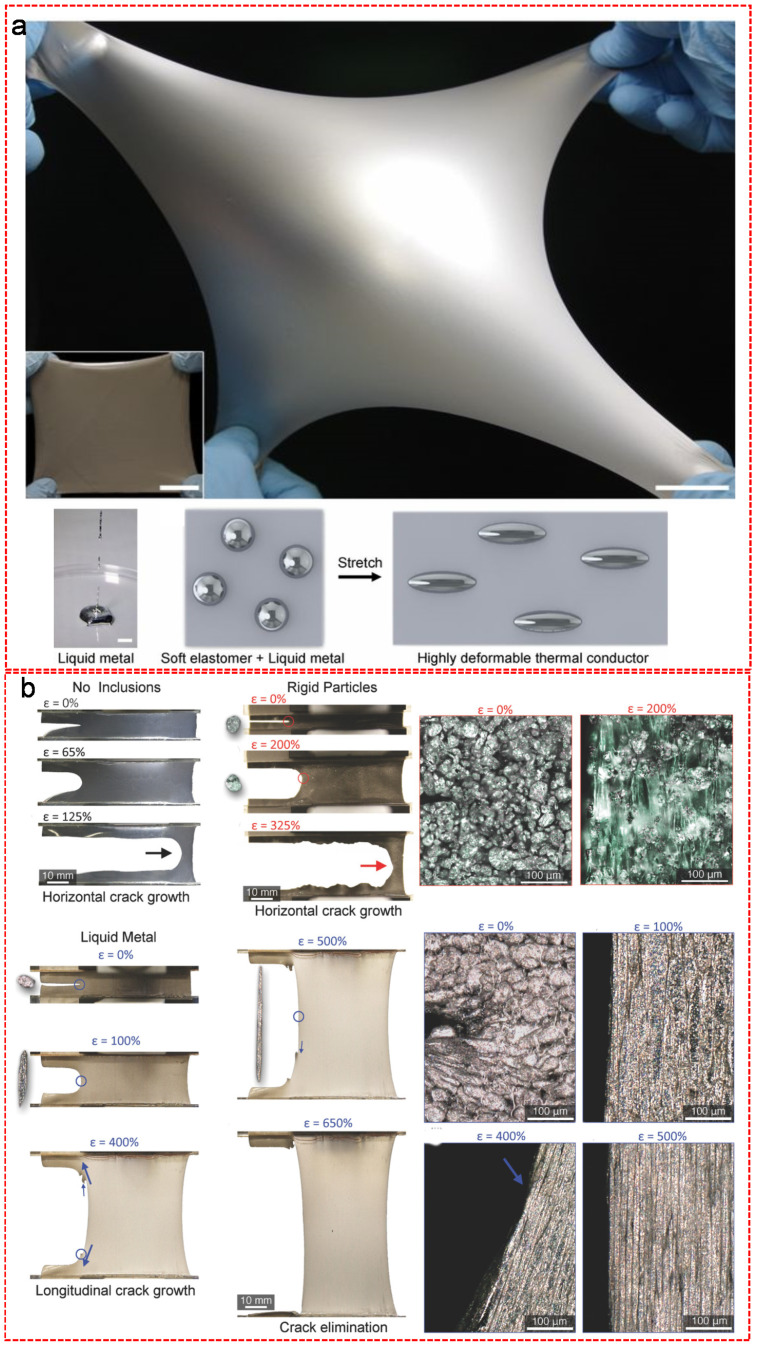
Synthesis of LM-polymer composites. (**a**) Highly deformable thermal conductor formed by LMEE. Reproduced with permission. [30] Copyright 2017, National Academy of Sciences. (**b**) Comparison of crack movement behavior among unfilled, rigid particles filled and LM filled polysiloxanes with different stretched states and their corresponding optical microscopy images. Reproduced with permission. [31] Copyright 2018, WILEY-VCH.

LM-polymer composites have been prepared by suspending nanoscale droplets of LMs or alloys as fillers in soft polymer matrixes to form biphasic compositions with tailorable electrical, dielectric, mechanical and thermal properties. Traditionally, rigid fillers such as rigid metal, ceramics or carbon-based materials have been employed to tune the properties of soft electronics. However, these traditional rigid fillers normally compromise the mechanical strength and deformability of polymer matrixes. LMs or alloys, as the conductive fillers, are able to solve these problems due to its excellent fluidity. Initially, LM-polymer composites were fabricated by the encapsulation of Ga-In LM alloy in the in situ formed urea-formaldehyde resin to function in a self-healing electronic circuit [28]. Afterwards, Mei et al. proposed a new concept called “LM filler”, which is contrary to the traditional “solid particle filler” [29]. In their work, the GaInSn LM alloy was directly mixed with methyl silicone oil in a beaker and then the mixture was stirred in air with an optimized rotation speed at 200 rounds per minute. The resulting LM poly-grease was obtained after a vacuum degas step to avoid the degradation of its thermal conductivity by air bubbles [29]. Subsequently, the “LM filler” was dispersed into a silicone elastomer, forming a highly deformable and thermally conductive LM embedded elastomer (LMEE), as shown in Figure 3a [30]. The resulting LMEE showed an unprecedented combination of excellent thermal conductivity (4.7 ± 0.2 W⋅m^−1^·K^−1^ under stress-free conditions; 9.8 ± 0.8 W⋅m^−1^·K^−1^ at 400% strain) with a low modulus (89 ± 2 kPa after 200% pre-strain) and high strain limit (>600%) [30]. The researchers compared the toughness of the unfilled elastomer and the ones filled with rigid particles or LM [31]. The unfilled sheet of silicone with a notch reached a complete failure with a maximum applied strain at 100% and the rigid particles filled one failed at an applied strain of 325%, while the LM inclusions elongated with the background strain field and consequently at an applied strain up to 650%, thus the LMEE remained intact with the initial notch eliminated due to the excellent fluidity of the LM inclusions (Figure 3b) [31].

**Figure 4:**
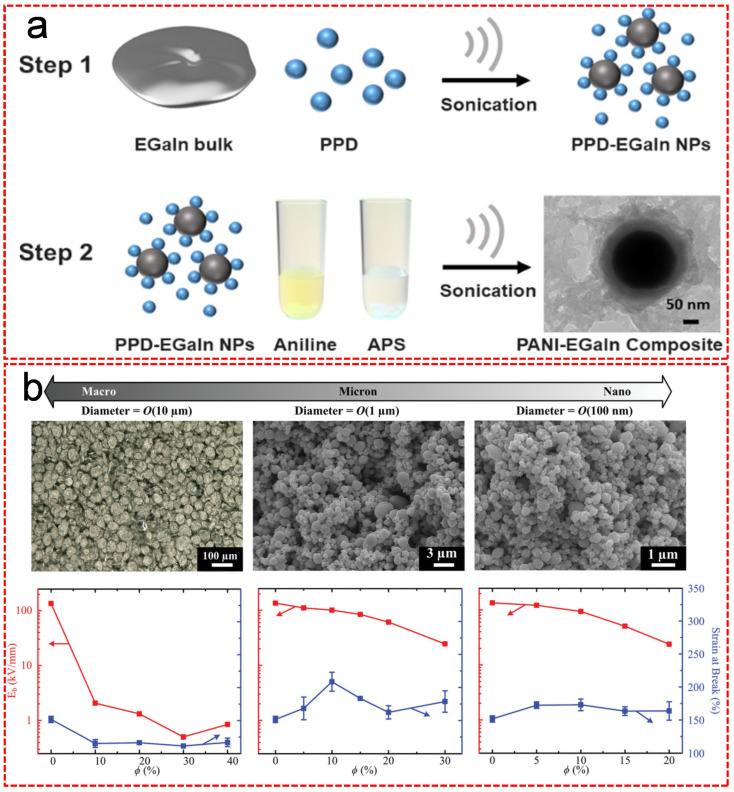
Synthesis of LM-polymer composites. (**a**) Synthesis of the PANI-EGaIn nanocomposites with the PPD polymerization enhancer. Reproduced with permission. [33] Copyright 2020, American Chemical Society. (**b**) The effect of sizes of LM particles on the dielectric and mechanical properties of the LM-polymer composites. Reproduced with permission. [34] Copyright 2019, WILEY-VCH.

Besides the direct mixing strategy, some other methods have also been proposed to form functional LM-polymer composites. Su et al. showed a simple method of synthesizing core-shell structured poly(n-butyl methacrylate) coated EGaIn composite (EGaIn@PBMA) via in situ radical polymerization [32]. In this study, EGaIn nanoparticles were functionalized with oleic acid (OA) as an environmentally friendly surfactant to form a complex. The introduced OA, on one hand, controlled the dispersion of the EGaIn nanoparticles and on the other hand, provided carbon–carbon unsaturated double bonds for the free radical polymerization with n-butyl methacrylate (BMA) monomers, in order to form a uniform PBMA shell outside of EGaIn core [32]. The soft core-shell structured EGaIn@PBMA nanocomposite showed excellent mechanical flexibility and dielectric properties. Zhang et al. proposed a two-step fabrication method of growing a polyaniline (PANI) nanofibrous network at LM nanoparticle interfaces for the generation of hybrid LM-polymer nanocomposites (Figure 4a) [33]. The bulk EGaIn was sonicated with the presence of p-phenylenediamine (PPD) in toluene as organic solvent to form dispersed PPD functionalized EGaIn nanoparticles, followed by the sequential addition of aniline and ammonium persulfate (APS) under sonication and ice bath to form the PANI nanofibrous network embedded EGaIn composite, which provided a road map for the direct synthesis of long organic molecular chains at the dynamic interfaces of LMs [33]. Furthermore, Pan et al. examined the impact of the size of the LM particles on the electrical and mechanical properties of the resulting composites [34]. As shown in Figure 4b, the nanoscale LM inclusions can enhance the electrical permittivity of the composite material with no significant reduction on the elastic compliance, stretchability or dielectric breakdown strength [34]. In contrast, the microscale LM inclusions can also improve the electrical permittivity of the composite material but with a dramatic reduction in breakdown strength [34]. Recently, a sedimentation technique was employed by Zhu et al. in order to fabricate functional LM-elastomer composites with electric, mechanical and thermal anisotropies [35]. In this research, Ga was firstly mixed with Polydimethylsiloxane (PDMS) to form a uniformly dispersed LM-polymer composite, followed by the sedimentation of the mixture in a mold for 10 min. After the composite was degassed and cured at 65 °C, the mixture separated into a Ga-rich layer pointing upwards and a PDMS-rich layer pointing downwards with electrical, thermal and mechanical anisotropy. Multilayer composite was achieved by repeating the above-mentioned processes on the former layer. Upon increasing the amount of Ga, the thickness of the Ga-rich layer can be expanded, which results in an improvement of the electrical conductivity and Young’s modulus of the composite but an unignorable decrease of the fracture strain. Interestingly, the composite is electrically and thermally conductive within the Ga-rich layer but insulating within the PDMS-rich layer or across the two layers.

The LM-polymer composites normally shows great stretchability and deformability, which are mandatory for the fabrication of LM-based soft electronics. However, the integration of LM with polymers usually sacrifices its intrinsic properties (e.g., excellent conductivity). Synthesizing highly conductive LM-polymer composites (e.g., highly conductive LM-elastic fiber) is an important research direction for improving the performance of LM-based soft electronics. Fabricating smart LM-polymer materials is of major interest (e.g., combining LM with shape-memory function). These will be discussed in the Section 3.1.

### 2.3. LM-Inorganic Nanoparticle Composites

**Figure 5:**
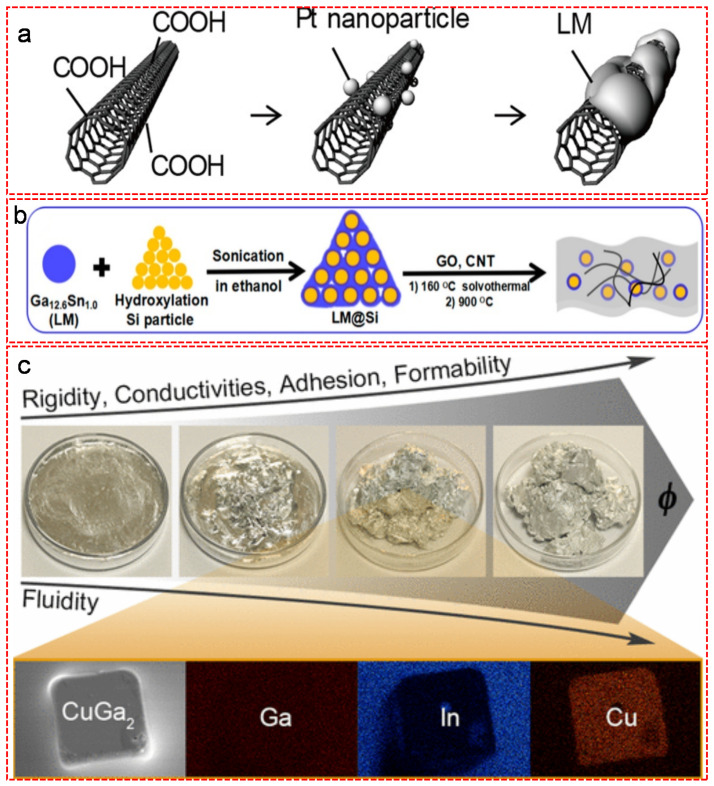
Synthesis of LM-inorganic nanoparticle composites. (**a**) Schematic illustration of the synthesis of CNT/LM composite by homogeneously dispersing CNTs modified with Pt nanoparticles in a liquid metal matrix. Reproduced with permission. [39] Copyright 2019, American Chemical Society. (**b**) Schematic illustration of the synthesis of Si/LM composite using CNTs and GO as the conductive network and skeleton. Reproduced with permission. [40] Copyright 2018, American Chemical Society. (**c**) The impact of Cu ratio on the physical properties of LM-Cu composite. Reproduced with permission. [41] Copyright 2017, American Chemical Society.

In order to enrich the functionalities of LM, doping with trace amount of inorganic nanoparticles such as carbon allotropies, ceramics and traditional rigid metals has been proven an effective strategy. Many methods have been applied to the incorporation of various nanoparticles into LM, thus forming LM-inorganic nanoparticle composites. The obstacle of forming LM-inorganic particle composites is the existence of the native oxide layer at the interface, which acts as a barrier to prevent inorganic particles from merging into LM. Prior to the formation of composition, the interfacial oxide layer must be removed. Zuraiqi et al. [36] and Daeneke et al. [37] demonstrated that acid-facilitated suspension, electrochemical amalgamation and mechanical mixing are the most commonly used strategies to effectively forming LM-inorganic nanoparticle composites or alloys. The acid-facilitated method use acid to remove the surface oxides of LM and metal particles, leading to the amalgamation of LM droplets with metal particles. The electrochemical amalgamation method is based on applying a substantial reductive potential to the metal particle pre-coated LM, which eliminates the oxides between the interface and thus promotes the amalgamation of metal particles into LM. The mechanical mixing method relies on destroying the interfacial oxides in the inert environment, thus ensures complete insertion of the inorganic particles into bulk LM. Ma et al. for the first time proposed the concept of the “nano LM fluid” and established an engineering route to design thermally conductive coolant by adding superior thermally conductive nanoparticles into the LM fluid [38]. The authors found that the particle size and shape, volume fraction and particle type played important roles in the enhancement of the thermal conductivity of LM-inorganic nanoparticle composites. Among those fillers, carbon nanotubes (CNTs) showed the best performance in the aspect of heat transfer. Following this track, Park et al. prepared a 3D printable soft electronic device with CNT/LM composite by homogeneously dispersing platinum (Pt) modified CNTs nanoparticles in a liquid metal matrix (Figure 5a) [39]. The CNTs were firstly functionalized with carboxylic groups, and then decorated with Pt nanoparticles as an interfacial material for binding CNTs and LM. Due to its high affinity to both CNTs and LM, the Pt nanoparticles was of great significance to avoid the separation and subsequent aggregation of CNTs and LM, leading to a uniformly dispersed CNT/LM composite. The resulting composite showed ultra-high mechanical strength in stretchable electronic devices. Wu et al. fabricated a Si/LM composite in corporation with CNTs and graphene oxide (GO) as a high-performance anode with self-healing function for lithium ion batteries (Figure 5b) [40]. The Si nanoparticles were firstly homogeneously mixed with LM by sonication in ethanol and milling to form a Si/LM composite, in which LM acted as a self-healing liquid buffer to restore the cracks caused by the volume expansion and contraction during lithiation and delithiation of Si. The addition of CNTs and GO worked as conductive network and skeleton to improve the conductivity of anode and prevent the Si/LM composite from traveling and aggregating. Besides carbon materials, rigid metal particles have also been incorporated with LM to alter their electrical, thermal, mechanical and magnetic properties. For instance, doping with different portions of Cu particles was confirmed as an efficient strategy to manipulate the physical properties of LM (Figure 5c) [41]. The resulting LM-Cu composite exhibited appealing semi-liquid/semi-solid mechanical behaviors such as excellent adhesion, tunable formability and self-healing ability, and with increasing the portion of Cu, remarkable enhancements in electrical conductivity (6 × 10^6^ S·m^−1^, ∼80% increase) and thermal conductivity (50 W·m^−1^·K^−1^, ∼100% increase) were recorded.

**Figure 6:**
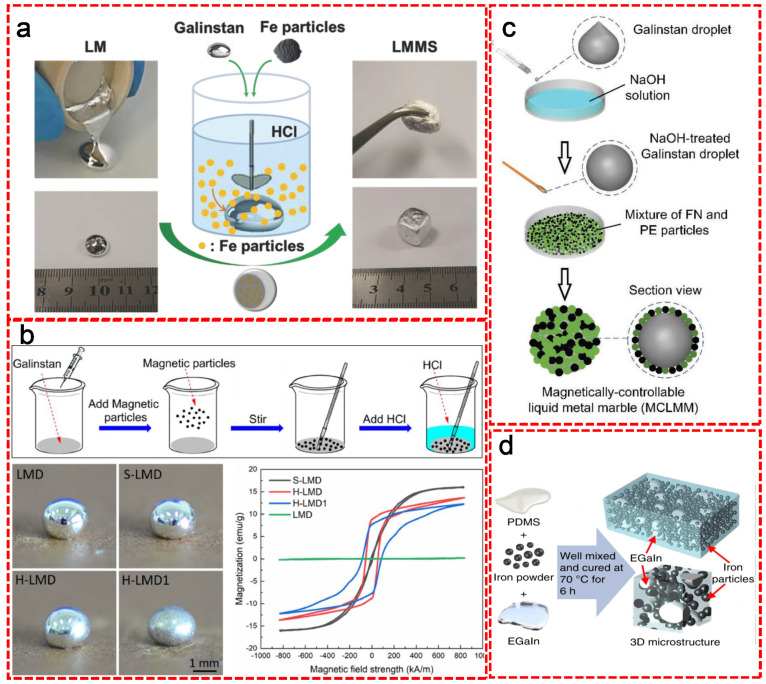
Fabrication of magnetically responsive LM composites. (**a**) Schematic illustration and photographs demonstrating the fabrication of LMMS. Reproduced with permission. [46] Copyright 2018, WILEY-VCH. (**b**) Schematic diagram of the preparation process of magnetic liquid metal. Optical images of the pure liquid metal droplet (LMD), magnetic liquid metal droplet with Fe particles (S-LMD), large-sized NdFeB particles (H-LMD1) and small-sized NdFeB particles (H-LMD). The magnetic hysteresis loops of the three kinds of MLMDs. Reproduced with permission. [47] Copyright 2021, Elsevier B.V. (**c**) Schematic diagram of the fabrication of the magnetically controllable liquid metal marble (MCLMM). A mixture of ferronickel (FN) and polyethylene (PE) particles was spread evenly in a Petri dish. Reproduced with permission. [48] Copyright 2019, WILEY-VCH. (**d**) Schematic of the procedure for fabricating the liquid metal-filled magnetorheological elastomer. Reproduced with permission. [49] Copyright 2019, Springer Nature Limited.

Moreover, particles bearing magnetic properties such as Fe, Ni and Co have been successfully added for preparing magnetically responsive LM composites [42,43,44,45]. Ren et al. reported a simple method for synthesizing LM-based magnetoactive slurries (LMMSs) by adding magnetic iron particles into a Ga-based LM matrix (Figure 6a) [46]. The native oxide layer of the Galinstan was removed in advance with diluted HCl solution, followed by mixing commercially pure Fe particles into Galinstan. Upon the addition of Fe particles, the liquid-state Galinstan was transferred into a readily patternable slurry-like material with a clear increase of viscosity. The successfully synthesized LMMSs exhibited a rapid response on mechanical properties, viscosity and stiffness when applying a magnetic field. He et al. showed a facial method of fabricating liquid metal composites with magnetic properties by simply adding different types of magnetic particles into Galinstan droplets (Figure 6b) [47]. After the mixture was welled dispersed by stirring, HCl solution was added to remove the oxide layer on the surface of the LM. Based on the type of magnetic particles, the resulting magnetic LM droplets (MLMDs) were classified into soft (with Fe) and hard (with NdFeB) MLMDs. The soft MLMDs can be easily manipulated under magnetic field due to the favorable deformability and flexibility, while the hard ones had much higher surface tension and mechanical robustness, as well as better electrical conductivity and strength. Chen et al. demonstrated the concept and fabrication procedure of magnetically controllable LM marble (MCLMM) (Figure 6c) [48]. A droplet of Galinstan was initially formed by squeezing raw LM alloy with a syringe in a petri dish, followed by a quick treatment using 30% NaOH solution to remove the oxide layer. As a result, a thin film of Ga-based gallate [Ga(OH)_4_]^−^ anti-oxidation layer was formed on the shell of LM droplet. After mixing with ferronickel (FN) and polyethylene (PE) microparticles, the sticky [Ga(OH)_4_]^−^ layer strongly bound to those particles, leading to the formation of MCLMM. The as-prepared MCLMM showed excellent magnetic controllability, good elasticity, favorable mechanical robustness and great stability in the ambient environment, which showed a bright potential in the field of robotic locomotion and manipulation, electronic circuits. With the help of PDMS matrix, Yun et al. fabricated a LM-filled magnetorheological elastomer comprising a hybrid of fillers of LM microdroplets and metallic magnetic microparticles by mixing iron powder with EGaIn and subsequent curing in the presence of PDMS at 70 °C for 6 h (Figure 6d) [49]. The resulting composite exhibited unconventional positive piezoconductive effect. In contrast to most elastic composites whose electrical conductivity decreased under tensile strain, the conductivity of the as-prepared composite was at a minimum level in the relaxed state and dramatically rose under strains. This exceptional phenomenon showed desirable potential for stretchable conductors. Moreover, the conductivity of the composite was highly responsive to the magnetic field. Upon applying an external magnetic field, the resistivity of the composite clearly decreased due to the reduced spacing of Fe particles arisen from the magnetic field induced particle alignment and the increased strain caused by the sample deformation.

## 3. Applications

Compared to the conventional rigid metals, LM offers better wettability, easier dispersion and deformation to conformably and non-destructively interact with the target substrate. Additionally, it is feasible to improve the interfacial contact resistance between the LM particles in order to create a conductive pathway by mechanical sintering at room temperatures [50,51,52]. The combination of their unique properties endows them with useful applications in various fields. In this section, we focus on discussing the potential applications of Ga-based materials in soft electronics, energy harvesting and storage materials, bio-medical applications.

### 3.1. Soft Electronics

**Figure 7:**
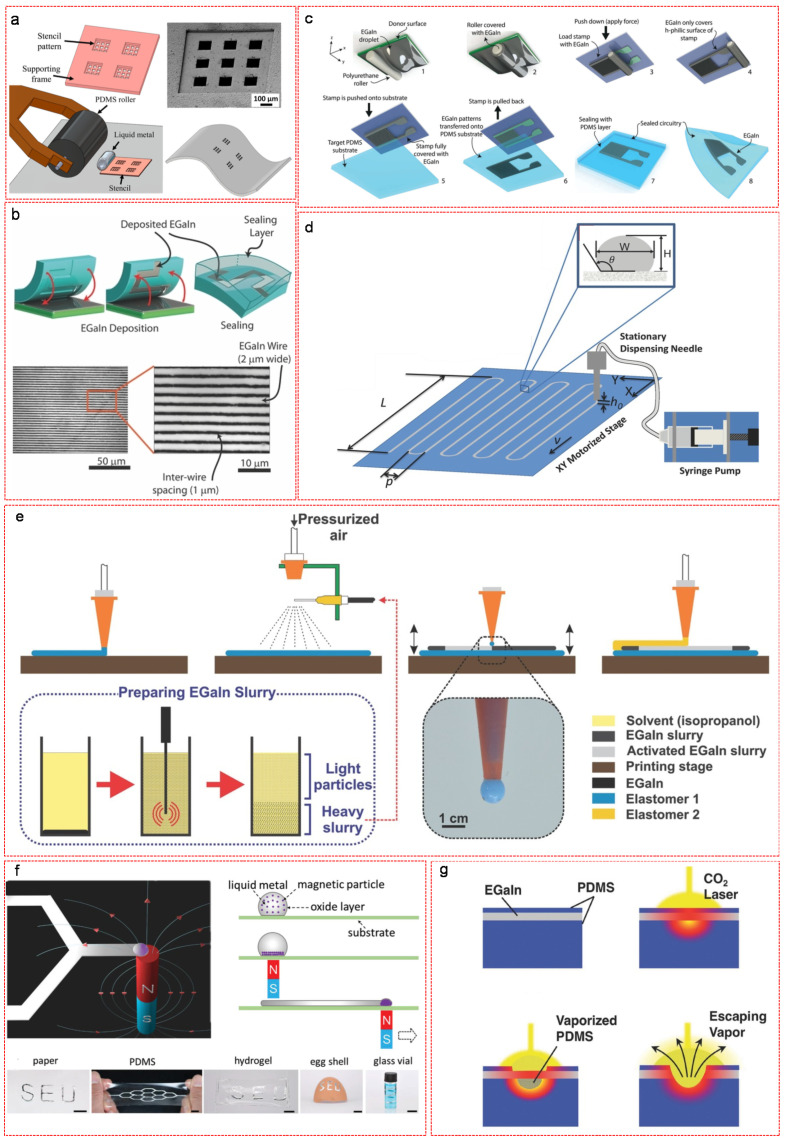
Feasible and efficient ways for LM patterning. (**a**) Stencil assisted printing for LM patterning. Reproduced with permission. [75] Copyright 2017, American Chemical Society. (**b**) Imprinting method for fabrication of LM microchannels with the resolution of 2 μm linewidth and 1 μm spacing. Reproduced with permission. [77] Copyright 2014, Wiley-VCH. (**c**) Microcontact printing process showing the fabrication and transfer of LM feature. Reproduced with permission. [78] Copyright 2019, Wiley-VCH. (**d**) Direct writing method for LM patterning using a stationary dispensing needle connected to a syringe pump and a xy motorized stage. Reproduced with permission. [79] Copyright 2014, Wiley-VCH. (**e**) The procedure of a fully automated printing for flexible and stretchable electronics by locally mechanical activation of LM. Reproduced with permission. [80] Copyright 2017, Wiley-VCH. (**f**) Magnetic field induced direct patterning of LM on various substrates. Reproduced with permission. [81] Copyright 2019, Wiley-VCH. (**g**) Schematic illustration of LM patterning by expulsion of a thin film of liquid GaIn alloy on a PDMS substrate with CO_2_ laser. Reproduced with permission. [82] Copyright 2014, Wiley-VCH.

The stretchable and patternable abilities are of great importance for materials used in soft electronic devices. The conventional rigid conductors are applicable to adapt the requirement for building soft electronics either by patterning the rigid metal trace with “deterministic geometries” [53] or by integrating conductive particles in flexible elastomer matrix [54,55,56]. In the former method, the rigid metals can be patterned into nanomeshes [57,58,59,60], wavy structures [61], serpentine interconnects [62] and fractally designed structures [63,64]. After patterning, the flexible trace layer can, to small extent, deform with the applied strain. However, the rigidity of the materials still largely compromises the ultimate strain at failure and the thin thickness often limits the electrical conduction. In the latter method, the conductive rigid particles (e.g., Cu [65,66], Ag [67,68], CNTs [69,70,71], graphene [72,73,74]) are well dispersed in elastomer matrix to create stretchable composites. However, the electrical conductivity of such composites is normally compromised by the non-conductive elastomer and the mechanical strength is highly affected by the rigid fillers. Contrarily, LM based materials, as intrinsically stretchable conductors, are suitable to build soft electronics, because they can maintain their bulk conductivity and mechanical strength during elongation under large strain. More importantly, both conventional and unconventional methods are practically operable for the patterning of LM based materials in order to fabricate highly thermally and electrically conductive, self-healing, durable and stretchable wires, interconnects, or antennas. Lazarus et al. applied a conventional method to creating LM 2D patterns (Figure 7a) [75]. In this case, the LM alloy was firstly casted on the surface of a multilevel Cu stencil which was fabricated by the method of masked electroplating with 3 μm patterns and 30 μm frame. Afterwards, the LM alloy was pressed and transferred into the cavities of the stencil, followed by repeatedly rolling the surface with a roller to ensure a uniform thickness of the LM and remove the excess of material. After removing the stencil, the LM patterns was fulfilled. The printing resolution was down to tens to thousands µm and the performance remained acceptable in an integrated device after stretching between 0% and 40% elongation. Park et al. further modified the method by using a photoresist as a mask to achieve a high-resolution and precisely aligned pattern of Ga-based LM down to 20 µm [76]. Imprinting methods are simple and effective alternative ways to produce desirable pattern features. Gozen et al. demonstrated an imprinting method for producing soft and stretchable Ga-based micron-scale electronics (Figure 7b) [77]. In this approach, an elastomer mold with micro-scale designed features was prepared via elastomer replica molding. Afterwards, LM alloy was uniformly spread over an elastomeric “donor” substrate with a roller, followed by removing the excess of LM material and flattening the surface with a flat elastomeric substrate under compression. Then, the elastomer mold was pressed onto the LM layer in order to fill the mold features. Finally, the patterned LM micro-channels were sealed with polymerized PDMS layers, functioning as stretchable circuit wires or capacitor electrodes. The printing resolution of such method reached 2 μm linewidth and 1 μm spacing. Yalcintas et al. modified this method by using micro-contact printing technique to reproducibly fabricate LM-based soft electronics (Figure 7c) [78]. A polyurethane roller was coated with LM by rolling from a donor substrate-supported LM surface, which subsequently loaded elastomer stamps with designed features. Then, the stamps were pushed onto target PDMS substrates and pulled back for the transfer of the LM patterns. The eventual sealing of LM patterns with another PDMS layer fulfilled the fabrication of LM soft circuitry. Direct writing method is a practicable way for the fabrication of LM circuitry with complex patterns. Boley et al. designed a direct writing system that allowed depositing serpentine patterns of LM materials on xy motorized stage by using a stationary needle that was connected to a syringe pump as the LM source (Figure 7d) [79]. Mohammed et al. modified this method and demonstrated a fully automated additive manufacturing process that produced all-printed flexible and stretchable electronics with LM slurry (Figure 7e) [80]. A base elastomer layer with optimum hardness and viscosity was initially deposited on a stage via extrusion printing using a nozzle, followed by spray printing of LM slurry to achieve consistent and uniform deposition of crack-free films. The selective activation of LM films was accomplished by mechanical contact with a pressure-applied nozzle via a tapping-mode operation of a working stage that moved up and down at a set frequency and simultaneously in x and y-directions to create desired conductive paths. Prior to the sealing of the device, conductive wires were connected to the terminals of the activated LM patterns. In addition, the magnetic field assisted method was proven a simple, versatile and equipment-free approach for direct patterning of magnetic LM particles on various substrates. Ma et al. demonstrated the concept of LM patterning by Magnetic field (Figure 7f) [81]. In this method, the magnetic LM/Ni microparticles were dropped on the target substrate. Upon applying a magnetic field with a magnet beneath the substrate, the Ni microparticles in the droplet were aligned at the bottom of the droplet. By applying a relative movement between the magnet and the substrate, the LM droplet was dragged by the aligned Ni microparticles to desired features. After getting rid of the remaining Ni particles and the excess of LM, a thin film of LM pattern was fulfilled. Based on the magnetic field enhanced contact between LM and substrate, this method was applicable to supermetallophobic substrates (e.g., paper). By remote manipulation of the magnetic LM, direct patterning on nonplanar surfaces (e.g., eggshell) or in a narrow and near closed space (e.g., the inner wall of a glass vial) was realized. Direct laser patterning is a rapid and inexpensive way for LM patterning. Lu et al. demonstrated the versatility of fabrication of electrically functional soft-matter sensors and circuit elements using a CO_2_ laser in minutes (Figure 7g) [82]. The principle of this method was simple. A LM layer was sandwiched by two PDMS layers to prevent oxidation and limit exposure. After exposed to CO_2_ laser (10.6 μm wavelength) the PDMS layers were locally heated and evaporated. When the surface tension of the LM film was surmounted by the pressure difference between the vaporized polymer and atmosphere, LM film was patterned through the locally vapor punctured LM materials.

**Figure 8:**
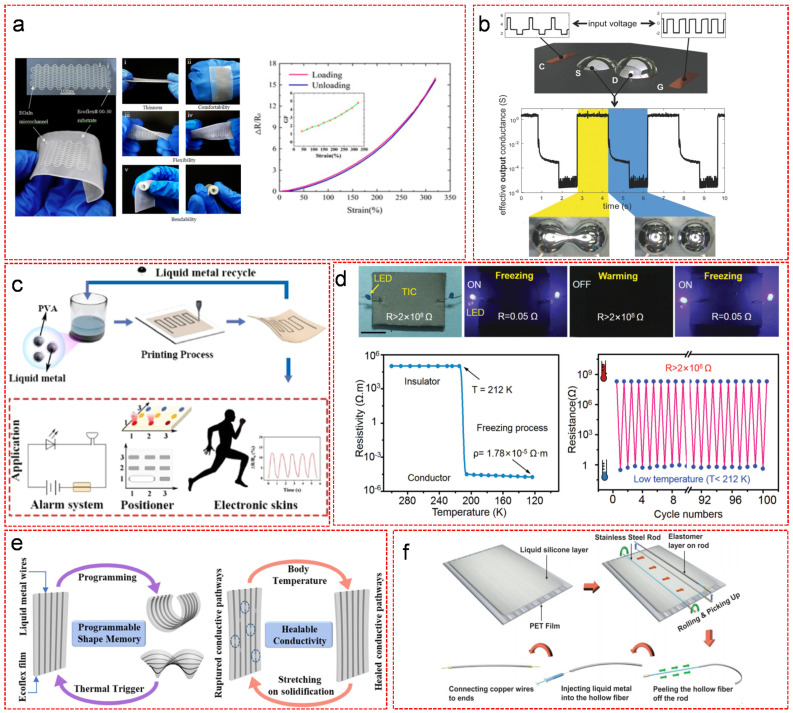
Soft electronics using LM based materials. (**a**) Optical image of the enhanced LM-based microfluidic strain sensor showing thinness, comfortability, flexibility and bendability, and its strain induced behavior of relative change in the resistance when stretched. Reproduced with permission. [85] Copyright 2021, American Chemical Society. (**b**) field-controlled electrical switch fabricated with LM as the source and drain materials. Reproduced with permission. [90] Copyright 2017, Wiley-VCH. (**c**) Printable and recyclable conductive LM ink for building alarm system, positioner and electronic skins. Reproduced with permission. [91] Copyright 2021, American Chemical Society. (**d**) LED built by LM-polymer composite showing temperature-induced reversible transition between insulator and conductor. Reproduced with permission. [96] Copyright 2019, Wiley-VCH. (**e**) Schematic illustration of soft and stretchable LM composites with thermal-induced shape memory and healable conductivity. Reproduced with permission. [97] Copyright 2021, American Chemical Society. (**f**) Schematic demonstration of the fabrication process of a hollow elastic fiber with liquid metal core. Reproduced with permission. [98] Copyright 2021, Wiley-VCH.

The most astonishing property of Ga-based materials is their high metallic conductivity under large strain. Dickey demonstrated that LMs are the only materials with high stretchability and conductivity compared to other representative stretchable conductors such as deterministic gold thin film, conductivity composites or ionogel [83]. By applying the afore-mentioned methods, LMs and their composites are readily utilized as inks to be printed or patterned for soft interconnects, sensors, transistors, stimuli-responsive electrical switches and self-healing conductors. However, precisely patterning Ga-based materials remains challenging due to the poor line edge roughness caused by the sticky nature of the instantaneously formed oxide layer on the surface of Ga especially when applying the methods such as imprinting, micro-contacting printing or stencil-assisted deposition [84]. Joshipura et al. suggested that injecting Ga into microchannels may be a good choice to achieve precise Ga pattern [84]. Oxygen-free encapsulation with other materials can avoid the instantaneous oxidation of Ga, which may also solve this problem. Chen et al. reported a microfluidic flexible strain sensor based on injecting LM into elastomeric microchannel matrix (Figure 8a) [85]. The microfluidic strain sensor showed many advantages including thinness, comfortability, flexibility and bendability. It managed to resist a maximum strain up to 320% with fast response, high sensitivity of strain change with a maximum gauge factor (GF = 4.91) at the 320% strain load, (Figure 8a) [85]. Recently, Liu et al. fabricated a printable conductor using EGaIn with high conductivity (2.06 × 10^6^ S m^−1^), extreme high stretchability (>1000%), negligible strain-induced resistance change, good cyclic stability (consistent performance over 1500 cycles) and excellent interfacial contact with rigid surfaces [86]. More importantly, the compatibility of EGaIn conductor with scalable manufacturing methods may suggest an inspiration of direct conversion of the printed circuit board (PCB) assemblies into stretchable circuit board assemblies (SCBAs) [86]. LM based materials have also been widely used in stretchable field effect transistors [87,88,89]. Wissman et al. reported a field-programmable transistor with LM as the source and drain electrodes (Figure 8b) [90]. Similar to the conventional field-effect transistors, the switch between on/off states of the LM transistor was under voltage control. Applying a voltage over the gate-source threshold voltage can trigger the coalescence of the LM source and drain electrodes or a voltage across the gate and counter electrodes can cause capillary bridge separation due to the geometrically constrained droplet deformation during electrochemical oxidation. The periodic input voltage generated periodic signal of effective output conductance. Xu et al. developed a poly(vinyl alcohol)/LM composite (PVA-LM) with high surface tension and excellent surface wettability on diverse substrates as a printable and recyclable ink for building alarm systems, object locators and electronic skins (Figure 8c) [91]. The alarm system was fabricated by connecting an external power supply, a light-emitting diode and a buzzer with PVA-LM printed circuit, which would be triggered upon pressing, rolling or sweeping the circuit. The PVA-LM printed circuit was also well functional in a 3 × 3 array positioner. Objects falling at corresponding areas would turn on the positioning lights. Soft electric skins could also be prepared using the PVA-LM ink to monitor the motions of the different parts of human body. Once sensing external stimuli, such as pressure, bending and stretching, the cross-sectional area decreased and the movement of electrons was blocked, resulting in an increase of electrical resistance that was proportional to the intensity of the stimuli. Besides the mechanical and electrical stimuli, magnetic field was also utilized as a powerful tool to trigger the on/off switch of a magnetic LM based circuit [92,93]. Joen et al. reported a magnetic-field-driven electrical switch based on the manipulation of magnetic LM slug in microfluidic channels [94]. The magnetic LM slug was prepared by coating HCl-treated LM with Fe particles. By dragging the magnetic LM slug within the microfluidic channels with a magnet, the LED lights were sequentially switched on. Thermal treatment is an effective way to manipulate LM for electrical switching [95,96]. Wang et al. prepared a highly stretchable LM-polymer composite, which showed electrical switching behavior of reversible transition between insulating and conducting upon thermal treatment (Figure 8d) [96]. The as-prepared LED connected to the LM-polymer composite based circuits was initially off with a high electrical resistance (R > 2 × 10^8^ Ω), because the stretchable silicone polymer prevented LM droplets from merging to create a conductive path at the room temperature. Upon freezing the LM-polymer composite by liquid nitrogen, the LED light was turned on with a low resistance (R = 0.05 Ω), because the silicone shell became extremely thin and rigid, allowing the rigid liquid metal droplets to expand and escape from the rigid polymer matrix and then connect with each other to form a conductive path. After warming up the LM-polymer composite, the polymer matrix became elastic and stretchable again and the LM droplets shrank to liquid state. The polymer matrix worked as enclosures to isolate the LM droplets, leading to a breakdown of the conductive path. It was worth mentioning that the temperature to trigger the electrical transition was about 212 K, at which point the resistivity value suddenly changed by more than nine orders of magnitude. Recently, Bhuyan et al. synthesized an elastomeric material with metallic conductivity and shape memory properties by injecting Ga into soft silicone microchannels (Figure 8e) [97]. On one hand, as a phase-change material, the rigidity of Ga helped the polymer to hold its programmed shape and store elastic energy in the external silicone matrix at the room temperature. On the other hand, the fluidic nature of gallium exhibited a rapid recovery (3−38 s) of the original polymer shape and the conductivity of Ga wires at high temperatures (35−65 °C). Sin et al. fabricated an ultrastretchable thermo- and mechanochromic fiber with healable metallic conductivity (Figure 8f) [98]. A stainless rod was covered with a composite of PDMS and thermochromic pigment by rolling-coating process. After thermal curing at 100 °C for 2 h, the solidified fiber was peeled off from the rod. Injecting EGaIn alloy into the hollow fiber and connecting Cu wire to the ends fulfilled the fabrication process. The fiber displayed the serial color change for the corresponding thermochromic pigments through precisely controlling the temperatures in the range 25 to 38 °C. Furthermore, the electrical conductivity of fractured fiber was successfully restored upon the introduction of body heat. Interestingly, Markvicka et al. fabricated an autonomously electrically self-healing LM-elastomer composite [99]. When the circuit was damaged, the LM droplets autonomously merged with neighbors to rapidly create new conductive paths and reconfigured around the damage in order to recover electrical signals with no interruption.

### 3.2. Energy Storage and Harvesting Devices

**Figure 9:**
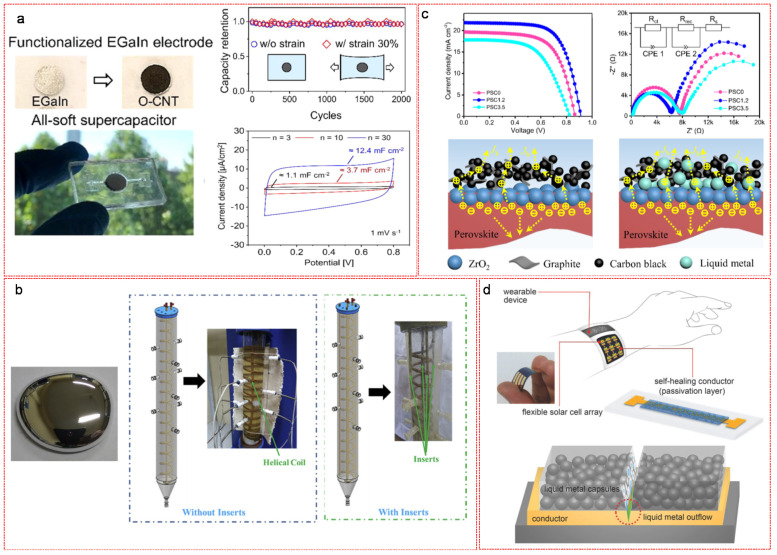
LM based materials for energy storage and harvesting devices. (**a**) All-soft LM supercapacitor showing an ultra-high capacitance and stable cycling performance under 30% strain. Reproduced with permission. [111] Copyright 2020, American Chemical Society. (**b**) LM inserted novel heat transfer enhanced solar thermal energy storage system. Reproduced with permission. [112] Copyright 2020, Elsevier B.V. (**c**) The mechanism of the interface engineering with LM for enhanced hole extraction and transport, and the corresponding current-voltage, electrochemical impedance curves. Reproduced with permission. [113] Copyright 2018, American Chemical Society. (**d**) Self-healing LM conductor for flexible wearable solar cell to restore the electrical conduction when damages happen. Reproduced with permission. [115] Copyright 2018, Wiley-VCH.

LM based materials have been widely used in energy storage systems (e.g., LM batteries [100,101] and capacitors [102]) and energy harvesting devices (e.g., wearable electric generators [103,104,105] and solar power systems [106,107]) due to their intrinsic fluidity, high thermal and electrical conductivities, nonflammability and non-toxic characteristics. More importantly, the self-healing ability of LM based materials can restrain the formation of Li dendrite growth caused by uneven nucleation and the crack of lithium ion batteries (LIBs) electrode caused by volume expansion/contraction during lithiation and delithiation reactions. Furthermore, LM can rapidly dissipate heat in photovoltaic cells to avoid fatal damage and dramatically enhance the heat generation efficiency and transfer efficiency of solar power generation and storage systems. Owing to their high boiling point and low melting point, LM based energy storage and harvesting systems can operate stably and safely at high temperatures, and maintain good interfacial properties at low temperatures. Li et al. demonstrated the advantages of using LM based electrode for batteries, compared to conventional solid metal and metal/alloy composite electrodes [108]. No Li dendrites were formed on LM electrode during the electrochemical process. In addition, LM can flow to eliminate the grain boundaries of electrodes during cycling in order to prolong the cycling life of batteries. Moreover, the interfacial contact between LM electrode and electrolyte was much better than the conventional solid electrode. In the earlier stage, Ga was directly used as a LIB anode for the purpose of improving the capacity and durability of electrode materials [109]. Compared to the conventional graphite anode, the theoretical columbic capacity of Ga (769 mAh·g^−1^) was doubled owing to the transition from Ga to Li_2_Ga during charging process. The Ga anode exhibited an excellent self-healing function during the charging and discharging process [109]. Upon lithiation, the Ga LM electrode crystallized to become a solid-state Li_2_Ga and during delithiation, cracks were formed and subsequently repaired based on the transformation of the solid-form Li_2_Ga back into a liquid-state Ga. However, a gradual decrease of capacity with cycling occurred, probably due to the instability of the solid electrolyte interface. Introducing other elements was proven a promising strategy to enhance the stability of the Ga anode. Wu et al. prepared a LM-based self-healing anode for LIBs with Ga-Sn alloy as the active material and reduced graphene oxide (rGO)/CNT as the skeleton [110]. Introducing Sn decrease the melting point of Ga so that the Ga-Sn alloy was endowed with self-healing function at room temperatures and the higher specific capacity (993 mAh·g^−1^) of Sn dramatically improved the capacity of the Ga (769 mAh·g^−1^) anode. The rGO/CNT skeleton separated the Ga-Sn particles from each other, avoiding the aggregation and detachment from the current collector during cycling. The resulting Ga-Sn electrode showed a high capacity (775 mAh·g^−1^) and superior cycling performance (nearly no capacity loss within 4000 cycles). As for LM capacitors, Kim et al. reported an all-soft LM supercapacitor with an ultra-high capacitance reaching 12.4 mF·cm^−2^ and 95% capacitance retention after 2000 charging and discharging cycles under 30% strain (Figure 9a) [111]. The LM electrode was fabricated by patterning EGaIn alloy with a PDMS stamp followed by coating EGaIn surface with functionalized CNT, which enhanced the interfacial adhesion in between in order to avoid the delamination of the soft electrode during the strain induced deformation. The LM based materials have also played an important role in the fabrication of energy harvesters including solar energy harvesting devices (e.g., the solar thermal power generation system and solar photovoltaic cell) and soft wearable power generators (e.g., thermoelectric, triboelectric, piezoelectric generators). In a solar thermal power generation system, the essential parts are heat adsorption, transfer and storage. Therefore, the heat transfer medium is of crucial importance, as it determines the heat transfer efficiency from the heat collector to the generator. The conventional heat transfer mediums (e.g., heat transfer oil or molten salt) can only stably function below 600 °C, which is much lower than the working temperature of a typical solar thermal power generation system, leading to the decomposition of the heat transfer mediums. In contrast, LM can operate under high heat flux at high temperatures, due to its high boiling point, high thermal conductivity, nonflammability and non-toxic characteristics. Salyan et al. reported a novel heat transfer enhancement technique for solar thermal energy system with Ga acting as thermal energy carrier (Figure 9b) [112]. The novel system consist of a vertical cylindrical shell, a helical coil and Ga metalinserts. The experimental results indicated that the addition of Ga significantly enhanced the thermal performance of the system. In a photovoltaic cell, LM based materials can also act as an interface material to tune the interfacial properties between solid electrodes [113,114]. Zhang et al. demonstrated the interface engineering between ZrO_2_ perovskite and carbon electrodes using Galinstan as a modifier to fabricate a compact-layer-free, fully printable solar cell (Figure 9c) [113]. Introducing a small amount of LM (1.2% by weight) remarkably increased the converted current density of the solar cell and significantly enhanced the interfacial contact properties by decreasing the carrier transfer resistance at the interface between ZrO_2_ and carbon electrodes. More importantly, it improved the hole extraction via increasing the hole transport channel and reducing the possibility of carrier accumulation and recombination due to the high conductivity and density of LM particles. In addition, there is an increasing demand of electrical repairing of solar cells, as they are often working in extreme conditions. LM based materials are promising for prolonging the durability of devices to build sustainable and flexible solar cells with self-healing function. Chu et al. presented an efficient method for the fabrication of flexible perovskite solar cell applying Ga_0.61_In_0.25_Sn_0.13_Zn_0.01_ LM-polymer composite as the self-healing reactant to restore the function of solar cell when damage occurred (Figure 9d) [115]. The LM-polymer composite was synthesized by encapsulating Ga LM alloy with in situ generated urea-formaldehyde, which subsequently was uniformly deposited as a passivation layer onto the interconnects of the as-built wearable solar cell. Once the electrical circuit was damaged, the LM capsules rapidly ruptured and flew to the disconnected sites, leading to an immediate recovery of electrical pathways. The experimental results indicated a rapid recovery of photovoltaic function with a power conversion efficiency retention of 99%.

**Figure 10:**
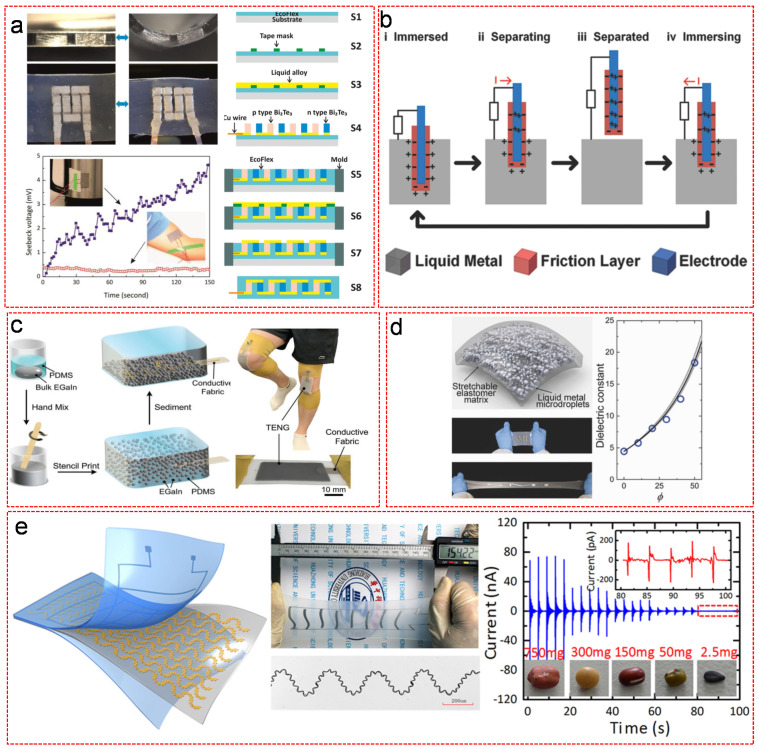
Various types of wearable energy harvesters based on different energy conversion mechanisms. (**a**) The schematic demonstration and photographs of a tailored layer-by-layer fabrication process of LM based highly stretchable, wearable TEG with high stability during mechanical deformation. Reproduced with permission. [119] Copyright 2017, American Chemical Society. (**b**) The working principle of LM based TENG. Reproduced with permission. [123] Copyright 2015, Wiley-VCH. (**c**) Ultrastretchable, wearable, skin-like TENG using sedimented LMEE as both the electrode and dielectric materials. Reproduced with permission. [124] Copyright 2020, Wiley-VCH. (**d**) The photographs of stretchable, high-k DEG using LMEE as the dielectric material. The dielectric properties of the LMEE are highly dependent on the fraction of LM. Reproduced with permission. [129] Copyright 2016, Wiley-VCH. (**e**) A hyper-stretchable self-powered PEG with 0.2 mg low detection limit and 1400 cycles’ stability test. Reproduced with permission. [138] Copyright 2017, Elsevier B.V.

Wearable energy harvesters that attain energy from human body have been of increasing importance for more complex and diverse tasks, which, based on the energy conversion mechanisms, can be classified into wearable thermoelectric generators (TEGs), triboelectric nanogenerators (TENGs), dielectric elastomer (DEGs) and piezoelectric generators (PEGs). For a better service, such devices have to possess merits such as reliability, durability, scalability, high power density and comfortability. For this reason, soft and stretchable electrode materials with excellent thermal, electrical conductivities, power conversion efficiency and biocompatibility are needed. As one of the most promising candidates, LM based materials have gained a lot of attentions owing to their unique properties. In typical wearable TEGs, thermal energy collected from human body heat was directly converted to electrical energy via the Seebeck effect in which current flow can be induced through the p- and n-type semiconductors by the temperature gradients between the skin of human body and the atmosphere. Many groups have reported the recent advances of using patterned LM [116,117,118], or LMEE [119,120,121] for wearable TEGs. Joeng el al. fabricated high-performance soft and stretchable TEGs using conventional rigid Bi_2_Te_3_ pellets metallized with Ga based LM alloy as the interconnect (Figure 10a) [119]. The device was built by means of a tailored layer-by-layer fabrication process. Initially, a thin film of 100 µm EcoFlex elastomer prepolymer was uniformly coated on a plastic substrate. After the EcoFlex elastomer prepolymer was semi-cured at 75 °C, a tap mask was placed on top for patterning Ga based LM alloy as the bottom interconnects. Following the removal of the mask, n- and p-type Bi_2_Te_3_ semiconductors were alternatively deposited on the top of the patterned LM alloy. An additional layer of LM alloy was sprayed on the semiconductors in order to improve their wettability. Afterwards, copper wires were connected to the LM interconnects, followed by filling the gaps of the patterns with the EcoFlex prepolymer in the help of a slide glass mold. After curing the elastomer at 75 °C, another LM layer was patterned as the top interconnects using a tap mask. Subsequently, the mask was removed, followed by sealing the device with a top layer of cured EcoFlex prepolymer. The device was completed after removing the plastic support substrate. The resulting stretchable TEGs exhibited an excellent room-temperature output power density of 40.6 μW/cm^2^. Upon introducing Ga LM alloy as the interconnects to the rigid pellets, the soft TEGs were still properly functional after being mechanically stretched and released more than 1000 times. Surprisingly, Malakooti et al. found that LMEE remained liquid state and maintained mechanical compliant and thermoelectric function at ultra-low temperatures, below −80 °C, which is much lower than the minimum working temperature of common fluidic LM systems [120]. To achieve this, carefully controlling the LM droplet size (to less than 3 µm in diameter) and appropriately selecting the polymer matrix were mandatary to strongly suppress their freezing temperature (down to −84.1 °C from −5.9 °C) and melting point (down to −25.6 °C from +17.8 °C) and thus maintained the mechanical compliant of the resulting LMEE based wearable TEG at the extreme low temperatures [120].

Flexible TENG is another type of wearable energy harvesters, which harnesses the biomechanical energies from human bodies by electrostatic induction between materials with different intrinsic tendencies to release or receive electrons according to the contact separation mechanism. Wearable TENGs have attracted considerable attention due to their lightweight, simplistic design, high efficiency and low cost. There have been four working modes of a TENG proposed based on the direction of the polarization change and electrode configuration, including vertical contact-separation mode, lateral-sliding mode, single-electrode mode and freestanding triboelectric-layer mode. The vertical contact-separation mode relies on controlled contact electrification caused by the relative motion of two dielectric electrodes perpendicular to the interface for the generation of potential change, which subsequently drives external current flow through the external load. In contrast, the lateral-sliding mode uses the relative displacement parallel to the interface. In a single-electrode mode, only one electrode (takes ground as the reference electrode) is normally used without attaching an electric conductor, while the freestanding triboelectric-layer mode takes a pair of symmetric electrodes as the reference electrode instead of ground. A superior TENG usually combines multiple types of working modes. The initial attempt of fabricating a TENG was done in the Wang’s group by using a paired unbonded polymer layers sandwiched by gold thin films with an output voltage of up to 3.3 V at a power density of ∼10.4 mW/cm^3^ [122]. Regardless its acceptable power density, the limited deformation range and the inefficient interfacial contact compromised its maximum power efficiency and durability. Therefore, the authors further exploited LM for the fabrication of a high-performance LM-based TENG [123]. The working principle was simple. One slice of friction material coated induction electrode was partially immersed into LM. Charges with opposite signs were stored on the surfaces of both friction layer and LM. When the induction electrode was lifted out of LM, the tribocharges were separated to induce a potential difference between two electrodes, thus the electrons were driven to flow through an external load (Figure 10b) [123]. Due to the liquid nature of LM, it ignored the roughness of the dielectric surfaces and allowed a total contact between the friction material and LM to enlarge the active contacting surface area, leading to a high output charge density of 430 μC·m^−2^ (4–5 times higher than that using a solid electrode), high power density at 6.7 W·m^−2^ and 133 kW·m^−3^, and an enhanced instantaneous energy conversion efficiency at 70.6% for Hg based TENG [123]. The authors further confirmed the concept of LM-TENG using pure Ga. The results showed that the Ga-TENG worked, suggesting that any other liquid metal materials could be utilized, such as commercially available Galinstan [123]. Owing to the instantaneous oxidation of Ga in the ambient condition, the oxides tended to stick to the friction materials, resulting in a partial screening of the tribocharges, and thus the performance of Ga-TENG was much lower than that of Hg-TENG [123]. The authors suggested that oxygen-free encapsulation of Ga should solve this problem [123]. In order to enhance its stretchability and durability, Pan et al. fabricated an ultrastretchable, wearable, skin-like TENG using sedimented LMEE as both the electrode and dielectric materials by forming phase-separated conductive and insulating regions (Figure 10c) [124]. The sedimated LMEE was simply prepared by mixing EGaIn into PDMS matrix with a wooden stirrer, followed by stencil printing onto a flexible conductive fabric. Then, reducing the temperature of the mixture to −16 °C for 15 min to slow down the curing of the matrix, promoting gravitational sedimentation of the LM inclusions. Eventually, the mixture was cured at 100 °C for 2 h. The wearable TENG exhibited excellent properties with ultra-thin thickness (around 1 mm), ultrahigh stretchability (>500% strain), skin-like softness (modulus < 60 kPa), reliable durability (>10,000 cycles) and acceptable electrical output performance (max peak power density at 1 mW·cm^−2^) [124]. Although many groups have devoted their efforts to improving the performance of TENGs by using LM materials, it remained unclear how the LM functioned in TENGs. Recently, research disclosed the effect of LM particles on the performance of TENG by incorporating LM particles into polyacrylonitrile (PAN) nanofibers [125]. When the LM content was small, the outputs of TENG rose with the increasing LM content. It reached a maximum output at about 1.5% LM content with the current density, output voltage and charge density increased by 40%, 70% and 70% respectively. A rapid degradation of the TENG performance happened upon further increasing LM content. The role of LM in the TENG was clarified that it enhanced charge trapping properties of the composite membranes to improve the performance but simultaneously the increased electrical conductance deteriorated the electrification effect. The former effect was dominant when the LM content was smaller than 1.5% by weight. However, the latter effect became dominant when the LM content was larger than 15% by weight, leading to a dramatic degradation of the TENG performance.

Different from the TENGs that generate electricity from friction and contact forces between dielectric elastomers, DEGs, subject to an applied voltage, convert mechanical energy from electromechanical coupling of dielectric elastomers to electric energy. Due to their low density, large actuation strain, rapid response and high specific energy density, DEGs have received much attention. The working principle of DEGs is simple. Thin films of dielectric elastomers were strained by external sources of force (e.g., wind, tide, human motions), followed by voltage induced charging process. After removing the external sources of force, the thin films of dielectric elastomers were relaxed so that the thickness of the elastomers increased and the surface area decreased, leading to an increase of charge density thus a potential difference. Finally, the accumulated charges were stored with a capacitor. In the early stage, a DEG was fabricated based on heel strike actuation using pure acrylic elastomer as the dielectric thin film to achieve 0.4 J/g specific energy density [126]. In order to enhance the electrical permittivity, pure elastomers have been filled with inorganic particles. Initially, rigid nanoparticles such as titanium dioxide (TiO_2_), Ni and Ag nanoparticles and CNTs were used as fillers to improve the dielectric properties, but they often compromised the strain limit of the elastomer matrix [127,128]. LM, as an alternative to the rigid fillers, can dramatically increase the electrical permittivity of LMEE thin film without degrading its mechanical properties. Bartlett et al. prepared stretchable, high-k dielectric elastomers by including different amount of EGaIn droplets in polymer matrix [129]. The resulting LMEE exhibited a low stiffness, high dielectric constant and large strain limit up to 600% (Figure 10d) [129]. The dielectric permittivity of the LMEE significantly increased with the increasing amount of LM, with a maximum four times (50% volume fraction of LM) higher dielectric constant than that without LM fillers. Despite that LMEE contributed to the excellent dielectric and mechanical performance of DEGs, it suffered from a low dielectric breakdown strength due to the large size and inhomogeneous distribution of the LM particles [129]. One promising solution is to use smaller sub-µm droplets instead of microsized ones, because smaller fillers can reduce the intensity of internal charge and field concentrations that lead to electrical breakdown [34]. Pan et al. systematically investigated the effect of LM droplet size on electrical permittivity, elastic compliance, stretchability and dielectric breakdown strength. The experimental results suggested that LMEE with smaller-size LM fillers (1 µm or 100 nm) enhanced its electrical permittivity without significantly compromising its elastic properties, stretchability, or dielectric breakdown strength, while LMEE with bigger-size LM fillers (10 µm) dramatically degraded its dielectric breakdown strength [34]. Tutika et al. confirmed that LM droplets with 1µm diameter were the ideal to achieve relative permittivity [130].

PEG that directly converts mechanical deformations (e.g., pressure, vibration, body motions) into electric energy is the prominent electromechanical energy harvester, owing to its high electromechanical coupling factor and piezoelectric coefficient compared to electrostatic, electromagnetic and triboelectric transductions. Furthermore, the piezoelectric effect is solely dependent on the intrinsic polarization of the material without the requirement of external stimuli such as a voltage source, magnetic field or contact with another material [131,132]. In addition, PEGs are durable, reliable, more sensitive to minor strains, and exhibit ∼3–5-fold higher density power output and higher voltage output compared to other energy harvesting devices [133,134]. Traditional piezoelectric materials are normally solid ceramics due to their high electromechanical coupling coefficient [135,136,137]. However, the stiffness and mechanical incompatibility hinder the rigid ceramic particles from direct incorporation into wearable PEGs. As with the aforementioned TEG, TENG and DEG devices, LM based materials have made a predominant contribution for the fabrication of flexible PEG to upgrade its stretchability, deformation detection limit and fatigue life. Huang et al. patterned EGaIn via electrohydrodynamic printing as microfluidic electrodes to acquire the accumulated charges from the strain-induced polarization of piezoelectric dipoles of self-similar serpentine-structured micro/nanofibers of PVDF in a hyper-stretchable self-powered PEG (Figure 10e) [138]. The as-prepared device displayed high stretchability (up to 320%), low detection limit (0.2 mg) and excellent stability under reciprocating deformation tests (1400 times at stretching 150%).

### 3.3. Biomedical Applications

**Figure 11:**
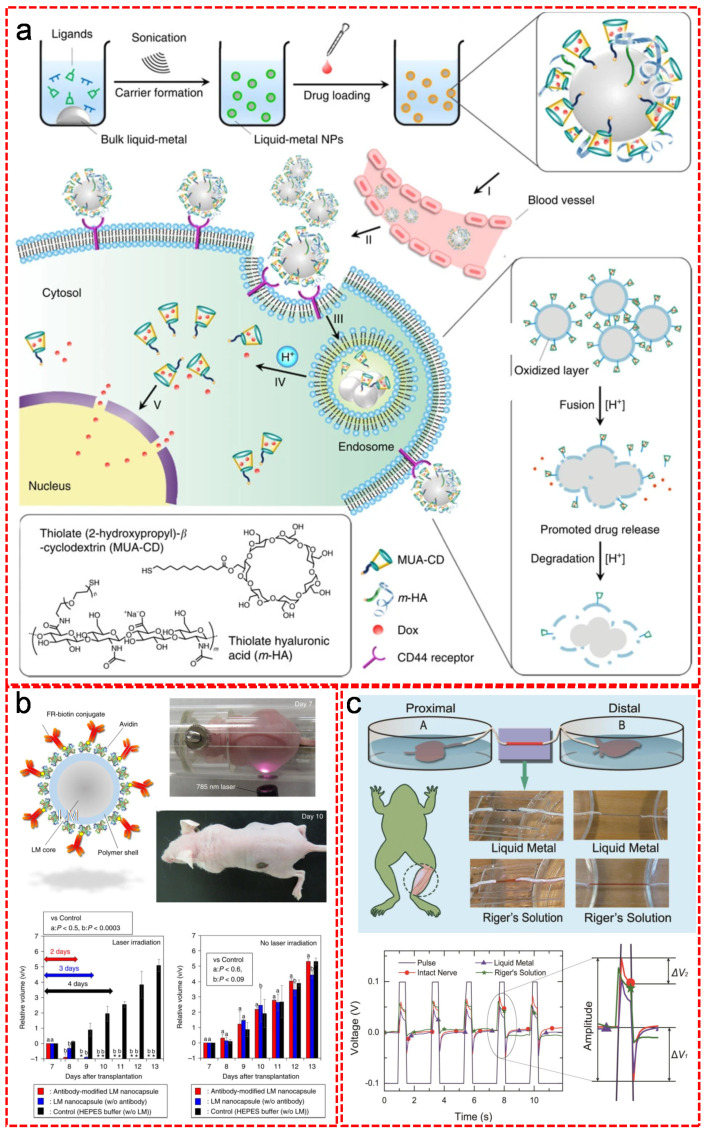
LM biomaterials for biomedical applications. (**a**) Schematic illustration of synthesis procedure of the transformable EGaIn nanomedicine and the working principle of drug delivery and release. Reproduced with permission. [140] Copyright 2015, Springer Nature Limited. (**b**) Functionalized LM capturing antibody for the rapid and effective tumor removal and corresponding data showing the comparison of time-dependent tumor volume change upon different types of treatment. Reproduced with permission. [151] Copyright 2017, Springer Nature Limited. (**c**) The transected sciatic nerve reconnected by the LM alloy showing a superior recovery of signal, close to that from the intact nerve. Reproduced with permission. [154] Copyright 2014, Zhang et al. (accessed on 19 July 2021).

LM biomaterials (especially Ga based materials) have become one of the most promising innovative functional materials to tackle modern biomedical challenges, owing to their many unconventional properties superior to traditional biomaterials, such as fluidic nature, low cytotoxicity, outstanding electrical and thermal conductivities, excellent biocompatibility, effective radiopacity, feasible processibility and cost-effectiveness [139]. Therefore, LM biomaterials offer more possibilities in the biomedical applications, such as drug delivery [140,141], tumor therapy [142,143,144], nerve repair [145], biosensing [146,147] and molecular imaging [148,149]. Lu et al. clearly demonstrated the working mechanism of using LM nanoparticles as nanomedicine carriers for drug delivery (Figure 11a) [140]. The LM drug nanocarriers were synthesized via the ultrasonication of bulk EGaIn in the presence of two ligands, thiolated (2-hydroxypropyl)-b-cyclodextrin (designated MUA-CD) and thiolated hyaluronic acid (designated m-HA) which controlled the size of LM nanoparticles. Furthermore, the former ligand worked as a drug-loading matrix (MUA-CD) and the latter one worked as an active targeting moiety (m-HA). Nanomedicine was prepared by loading the CD cavities with the drug molecule doxorubicin (Dox). After injecting the nanomedicine into blood vessel, they accumulated at the tumor sites based on passive and active targeting effects with the help of the interaction between m-HA moieties and CD44 receptors. In endosome, the acidic environment resulted in the fusion of LM particles and subsequent dissociation of the drug-containing ligands, promoting drug release. In addition, the main degradation product Ga (III) was formed via the synergistic effect of oxidative corrosion in the acidic environment, which acted as anticancer agent to reverse drug resistance. The drug releasing can also be triggered by light irradiation. Lu et al. [150] and Chechetka et al. [151] proved that near infrared light irradiation effectively induced the structure transformation of the LM based nanomedicine for spatiotemporally controlled intracellular drug delivery. Thanks to their remarkable thermal and electrical properties, LM biomaterials have also been exploited for curing tumors. Chechetka et al. synthesized epidermal growth factor receptor (EGFR) antibody functionalized EGaIn by coating the EGaIn with a polymer shell that strongly bound with avidin for capturing biotin-conjugated EGFR monoclonal antibody (Figure 11b) [151]. Compared to the conventional ones, the resulting LM biomaterial exhibited more rapid and complete removal of tumor upon photothermal therapy after only 3 days’ treatment [151]. The temperature change of EGaIn nanocapsules were totally controllable and no obvious side effects were observed [151]. Manufacturing LM into transformable electrodes was proven an efficient way for the electrochemical treatment of tumors. The conventional electrodes suffer from their rigidity that limits the effective therapy area, while LM electrodes can transform to adapt complex physiological situations for enhanced therapeutic efficacy [142]. LM biomaterials have also been used as contrast agents or radiotracers for medical imaging such as X-ray imaging, computerized tomography (CT) and photoacoustic imaging (PAI), due to their low toxicity, good biocompatibility and biodegradability. For instance, EGaIn-based electrodes can generate high photo-energy, leading to a deep penetration through thick tissues, thus high resolution and absorption-contrast imaging in angiography and mammography. Wang et al. clearly showed the comparison of the contrast effects between angiograms with liquid metal and conventional agent under X-ray irradiation [152]. The X-ray images clearly indicated that resolution was enhanced by LM reaching a detailed width of 0.1 mm for the tiny vessels, which was quantitatively supported by the grayscale histograms and numerical indexes. Larsson et al. further optimized the maximum brightness of heated EGaIn electrodes as X-ray sources to achieve 24.2 keV by increasing the relative fraction of indium up to 65% by weight in order to enhance the penetration depth and allow imaging of thicker samples [153]. LM biomaterials have also been explored as reconnection agents for peripheral nerve injury, because they were able to not only physically repair the nerves but also recover the electrical signal of the reconnected verves close to those from healthy ones. Zhang et al. for the first time proposed to use GaInSn LM alloy as connecting or functional recovery channel to repair the damaged peripheral neurotmesis (Figure 11c) [154]. The experimental results disclosed that the GaInSn LM alloy surpassed the conventional Riger’s Solution in many aspects as the nerve connector. Compared to the Riger’s Solution, the GaInSn showed an ultra-low impedance (serval orders lower) for the conduction of the weak electroneurographic signal. More importantly, the LM reconnected sciatic nerve exhibited a close electroneurographic signal to that from an intact sciatic nerve. In addition, the LM alloy was clearly visualized under the plain radiograph, which was a great convenience for surgery. LM biomaterials have also been of great convenience as skin-like biosensors for real-time monitoring of human health. Li et al. presented a LM based wearable, stretchable pulse sensor for monitoring patient’s heartbeat [155]. The core pulse sensor was fabricated by embedding LM functional circuits with PDMF layers. A wireless heartbeat monitoring system was developed by integrating the pulse sensor with a Bluetooth module, an Arduino development board, a laptop computer and a self-programmed visualization software program. Stable performance was observed from the stimuli induced voltage–time curve under various circumstances.

## 4. Conclusions and Perspectives

In this review, we summarize the recent advances of the synthesis of Ga based materials and their wide-range applications including soft electronics, energy storage and harvesting devices and biomedical materials. Low-melting-point LM materials possess a series of unique properties such as fluidity, self-healing ability, outstanding thermal and electrical conductivities and biocompatibility. So far, many methods have been developed to fabricate LM droplets and further control their size for the use towards different purposes. Due to its fluidic nature, LM materials can be readily manufactured as conductive ink to be printed on a wide range of substrates and easily patterned by diverse ways for the production of soft electronics. It has also played an important role in the development of self-healing electronic devices and batteries to maintain their performance due to mechanical damage or electrode failure during cycling. In addition, due to its high biocompatibility, LM have also been used for bio-applications. For example, the deformation induced phase transition of LM can promote rapid and efficient drug release in human body. Furthermore, their effective radiopacity and high thermal conductivity grant LM materials as contrast agent or photothermal electrode for medical angiogram or removal of tumor. In order to facilitate the functionalities of LM, composition strategy by integrating with polymers and/or inorganic nanoparticles have been proven an effective way to endow LM with more merits [156]. In contrast to those rigid metals, LM can easily fuse with other materials by mixing in ambient conditions to generate new heterostructures with controlled properties, such as enhanced thermal, magnetic and mechanical properties. The diverse LM functional materials dramatically widen the application range. For instance, LMEEs have been widely used in the fabrication of ultra-stretchable, printable electronic devices including wearable energy generators, solar cells and healthy monitors. LM-inorganic nanocomposites have shown great potential for heat absorption and transfer, robotic locomotion and manipulation. Highly stretchable Ga-polymer fiber has shown a bright future to complement or even replace the conventional Cu wire in many fields such as wearable electronics, soft robotics.

Despite the above-mentioned exciting advances, many challenges remain for further improvement. The greatest concern of utilizing LM based materials for bio-application is their controversial toxicity towards human body. Although compared to other LMs (e.g., mercury), Ga and Ga based materials have displayed good biocompatibility and low toxicity based on the in vivo experimental finding in animals, limited studies of acute and chronic toxicology have been conducted to gain comprehensive understanding about the toxic effects of Ga based materials in the complex human body environment. In addition, the size of LM nanoparticle strongly influences not only the physical properties but also the physiological behavior of LM materials, which brings more complexity into the research. Therefore, more advanced synthetic methods for the production of LM nanoparticles with controllable size and distribution must be developed, and intensive and extensive research of toxicology of LM have to be evaluated.

In another aspect, Ga is highly susceptible to the surrounding environment to form an ultra-thin layer of oxide. The native oxide layer of Ga has both advantages and disadvantages. From the positive point of view, it can help Ga control its shape and size and form a core-shell structure for further functionalization. Tang et al. considered the Ga native oxide layer as a platform for enhanced reactivity and systematically demonstrated the function of electrochemically driven reversible transition between Ga and Ga oxide layer on a wide range of reactions such as energy harvesting, soft robotics, field-effect transitors, soft diodes and memristors [157]. Liu et al. also demonstrated in detail the surface engineering strategy by synthesizing core-shell LM materials for improved performance in several attractive scenarios including but not limited to soft electronics, nano/biomedicine, catalysis and energy storage/conversion [158]. From the negative point of view, the existence of such oxide layer largely comprises fluidity and electrical conductivity of Ga. Moreover, the high stickiness of the oxide layer complicates the printing process of LM based soft electronics. Therefore, an efficient and convenient method of suppressing the formation of Ga oxide layer is urgent. Encapsulating Ga with other materials to form an anti-oxide shell may avoid the instantaneous oxidation, which may simplify the processing of Ga and achieve high patterning resolution. Realizing precise patterning and scaling-up manufacturing of Ga based materials would boost the development of Current technology of electronics.

Another problem is its high-temperature material compatibility. As an excellent cooling agent, Ga based materials have been popularly used for rapid dissipation of heat from systems in order to protect them from burning. However, at high temperatures, Ga-based materials themselves tend to corrode and embrittle other metal substrates (e.g., stainless steel, Al, Cu), causing possible failure of electronic devices, solar cells or heat storage systems. Although surface modification of LM nanodroplets can lead to stable anti-corrosion effect towards metals, it dramatically reduces its electrical conductivity. Therefore, more efforts have to be devoted to fundamental research about Ga corrosion mechanism and protection methods to explore practical coating strategy for effective metal protection without the degradation of electrical properties.

Although the electrical conductivity of Ga is sufficient for building electronic devices, it is still one magnitude lower than Cu. Therefore, further improving the conductivity of Ga based materials is of critical importance to enhance the performance of Ga based devices [159].

Currently, most of the research about Ga based materials have been limited in the lab. There is an urgent necessity for a smooth transition of the experimental findings to industries. For example, the safety issue and mileage anxiety of LIBs have always been huge obstacles for the development of electric vehicles. The current investigations have shown a promising future of solving those problem in the mild experimental conditions using the transformable Ga based LM materials to avoid the penetration of separator by the formed Li dendrite and suppress the expansion and damage of anode. However, the real scenario is more complicated with a great number of variables. Thus, more research must be done according to the strict testing standards.

In spite of those aspects to improve, the fruitful progresses of Ga based materials in recent years have showed a bright future of this new-generation functional materials. We strongly believe that with many unique properties Ga based LM materials will boost in all sorts of emerging research fields and industrial applications to facilitate people’s daily lives, innovate medical treatment, alleviate energy crisis and promote robotic artificial intelligence.
